# Breeding perspectives on tackling trait genome-to-phenome (G2P) dimensionality using ensemble-based genomic prediction

**DOI:** 10.1007/s00122-025-04960-6

**Published:** 2025-07-04

**Authors:** Mark Cooper, Shunichiro Tomura, Melanie J. Wilkinson, Owen Powell, Carlos D. Messina

**Affiliations:** 1 Queensland Alliance for Agriculture and Food Innovation, The University of Queensland, Brisbane, QLD 4072 Australia; 2 Australian Research Council Centre of Excellence for Plant Success in Nature and Agriculture, The University of Queensland, Brisbane, QLD 4072 Australia; 3 UF/IFAS Crop Transformation Center, University of Florida, Gainesville, FL USA

## Abstract

**Key message:**

**Trait Genome-to-Phenome (G2P) dimensionality and “breeding context” combine to influence the realised prediction skill of different whole genome prediction (WGP) methods. Theory and empirical evidence both suggest there is likely to be “No Free Lunch” for prediction-based breeding. Ensembles of diverse sets of G2P models provide a framework to expose and investigate the high G2P dimensionality of trait genetic architecture for WGP applications. Artificial Intelligence and Machine Learning (AI-ML) prediction algorithms contribute novel trait G2P model diversity to ensemble-based WGP. Prediction-based breeding leveraging ensembles of G2P models creates new opportunities to identify and design novel paths for genetic gain.**

**Abstract:**

Improving our understanding of trait genetic architecture is motivated by creating new opportunities to enhance breeding methodology, create new selection trajectories for crop improvement, and accelerate rates of genetic gain. With access to high-throughput sequencing, phenotyping and envirotyping technologies we can model the complex multidimensional relationships between sequence variation and trait phenotypic variation that are under the influences of selection. Using the framework of the diversity prediction theorem, we consider applications of ensembles of diverse trait genome-to-phenome (G2P) models. Crop growth models (CGM) are an example of a hierarchical framework for studying the influences of quantitative trait loci (QTL) within trait networks and their interactions with different environments to determine yield. Hybrid CGM-G2P models combine elements of CGMs, to understand how trait networks influence crop yield performance, with trait G2P models, to understand influences of trait genetic architecture on selection trajectories. We discuss hybrid CGM-G2P models and their potential applications to enhance ensemble-based prediction. Multi-environment trials conducted across breeding cycles can be designed to include contrasting environments to expose the different CGM-G2P dimensions of the trait by environment interactions that are influential on selection trajectories. Artificial intelligence and machine learning (AI-ML) algorithms can be applied as components of ensembles to improve gene discovery and quantification of allele effects for traits to enhance G2P prediction applications. We use the trait flowering time in the maize TeoNAM experiment to illustrate and motivate further investigations of how to leverage ensembles of G2P models for prediction-based breeding.

## Introduction

**Fig. 1:**
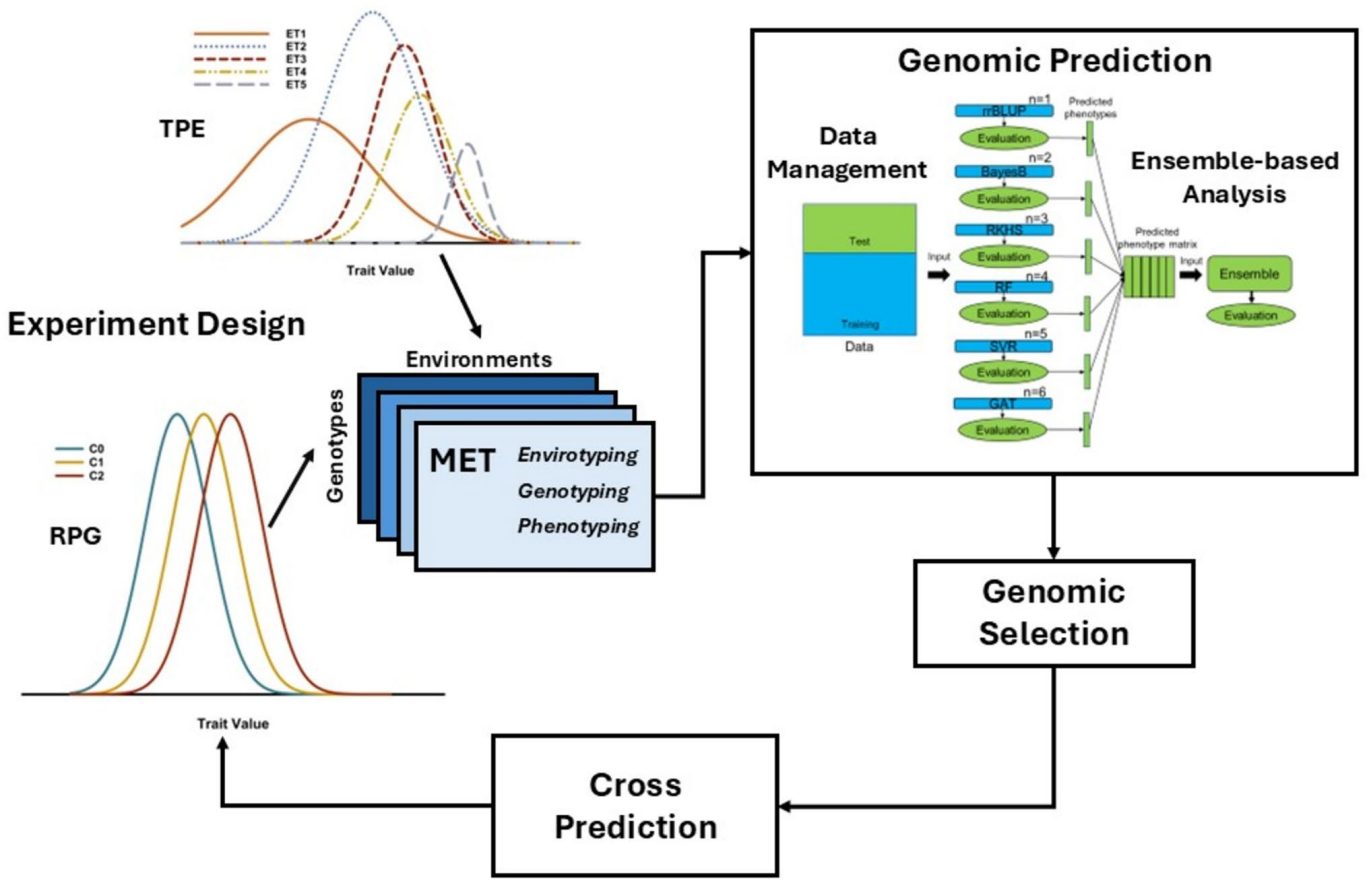
Schematic representation of a breeding program cycle, highlighting the application of an ensemble-based analysis of the data generated from a multi-environment trial (MET). METs are designed for all stages of a breeding program to collect trait phenotypic data based on an evaluation of samples genotypes from the reference population of genotypes (RPG), for the different stages of the breeding program, within samples of environments from the target population of environments (TPE). The trait phenotypic data are used in combination with genotyping data (e.g. single nucleotide polymorphisms SNPs) generated for the entries included in the MET to train whole genome prediction (WGP) models for use individually or within ensemble-based approaches. The MET data obtained from phenotyping and genotyping can be augmented with environmental data to characterise the environments and identify the representation of the envirotypes (e.g. ET1 for envirotype 1) within the TPE to account for genotype-by-environment-by-management (GxExM) interactions for traits

Perspectives on novel opportunities for design of ensemble methods for prediction-based breeding are discussed in relation to our current views of trait genetic architecture and experience gained from applications of prediction-based methodologies to crop breeding (Fig. 1; Hammer et al. 2006, 2019; Voss-Fels et al. 2019a; Cooper and Messina 2023). This background frames components of trait genetic architecture that can be targeted for inclusion within the model-ensemble. Following an introduction to potential sources of information to be included in ensembles, and the motivations and targets for constructing model-ensembles, we consider some key results from an application of a model-ensemble investigation of the TeoNAM experiment (Chen et al. 2019; Tomura et al. 2025a). Finally, we discuss some areas for further investigation and potential future directions for prediction-based breeding.

**Fig. 2:**
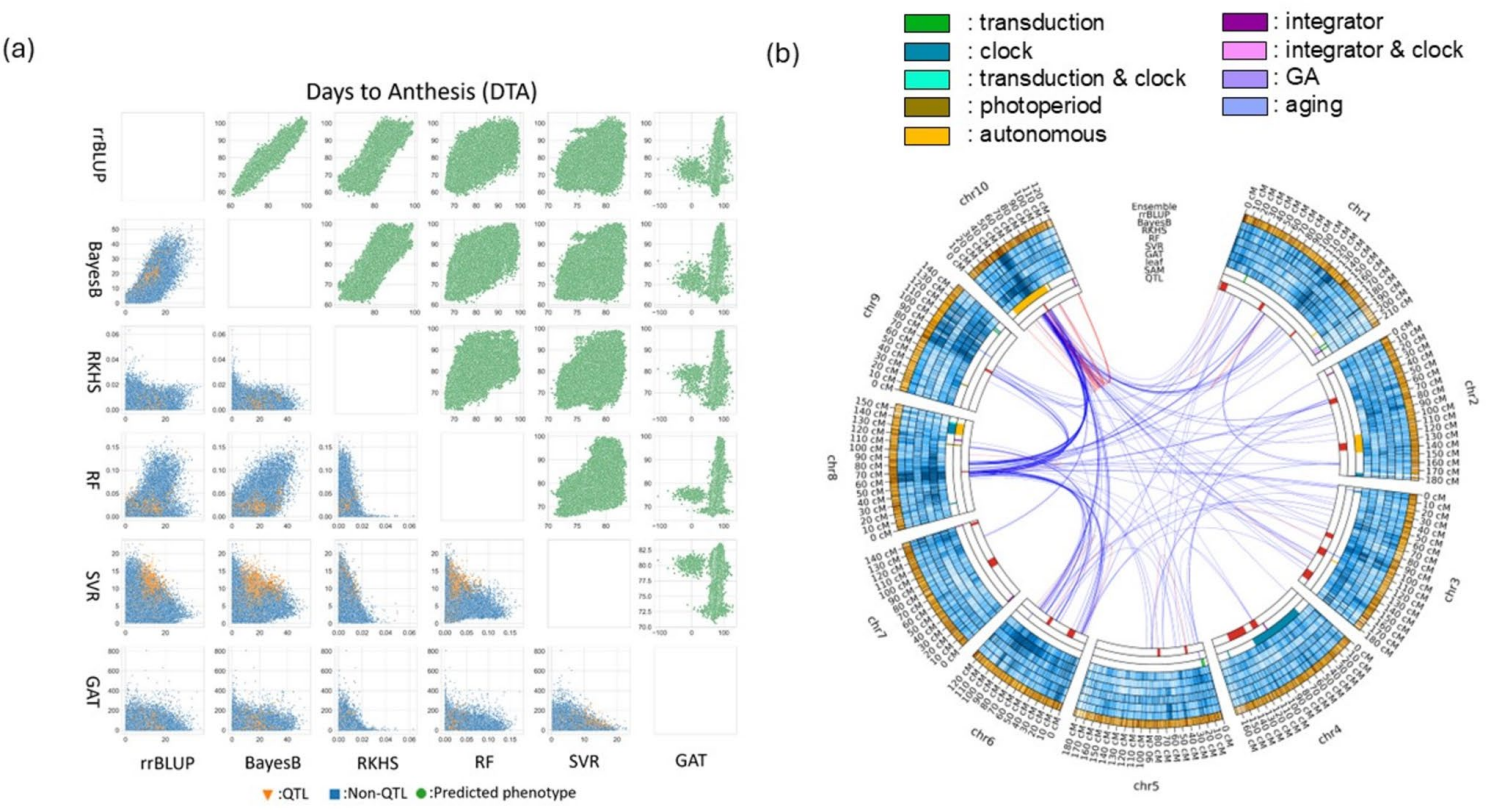
Example of an ensemble-based whole genome prediction (WGP) analysis of the TeoNAM experiment (Chen et al. 2019), based on the results of Tomura et al. (2025a): **a** Scatter diagrams comparing predicted total genotypic value, upper triangle, and single nucleotide polymorphism (SNP) effects, lower triangle, for application of six WGP models to the days to anthesis trait; the six models are ridge regression BLUP (rrBLUP), BayesB, reproducing kernel hilbert space (RKHS), random forest (RF), support vector regression (SVR), graph attention network (GAT). For the comparisons of the SNP effects, individual SNPs identified at the peak of quantitative trait loci (QTL), by Chen et al. (2019), are indicated in gold, all other SNPs are indicated in blue. **b** Circos plot (Tomura et al. 2025b) views of the genome positions and relative sizes of SNP effects obtained from application of the six WGP models to the days to anthesis trait measured on the TeoNAM experiment (Chen et al. 2019). Progressing from the outer ring inwards, the first ring is the ensemble followed by the six WGP models displayed individually; rrBLUP, BayesB, RKHS, RF, SVR and GAT. The next two rings progressing further inwards provide genome positions of genes and QTL included in a network model for the control of flowering time in maize, following Dong et al. (2012); light transduction, circadian clock, photoperiod transduction pathway, autonomous pathway, aging pathway, gibberellic acid (GA) pathway, Pathway Integrator. The innermost ring gives the genome positions of the QTL for the trait days to anthesis reported by Chen et al. (2019). Within the centre, the lines connect SNP genome positions to indicate putative interactions between SNPs based on the top 0.005% of shapley scores between all pairs of SNPs from the RF analysis; red lines indicate putative Cis-Acting SNP interactions. Blue lines indicate putative Trans-Acting SNP interactions

The central question we consider is “how to use whole genome prediction (WGP) to create novel selection trajectory outcomes from crop breeding” (Fig. 1; Podlich et al. 2004; Hammer et al. 2006; Messina et al. 2011, 2025; Cooper et al. 2005, 2014a,b, 2021a; Wisser et al. 2019; Technow et al. 2021; Powell et al. 2022; Choquette et al. 2023; Werner et al. 2025; Polzer et al. 2025; Escamilla et al. 2025). While this is a challenging ambition, recent results from applications of ensembles of prediction models indicate promising new areas that encourage further investigation (Bian and Holland 2015; McCormick et al. 2021; Kick and Washburn 2023; Fradgley et al. 2023; Heilmann et al. 2023; Kawakita et al. 2024; Washburn et al. 2025; Tomura et al. 2025a; Messina et al. 2025). Applying the framework of the “Diversity Prediction Theorem” (Page 2010, 2018; Messina et al. 2025; Tomura et al. 2025a), we review background and motivations relevant to applications of ensembles of diverse prediction models to create new opportunities to enhance WGP outcomes for crop breeding applications. The ensembles we consider herein are constructed by combining diverse sets of genomic prediction models that can be created from the types of data generated from breeding multi-environment trials (METs). These diverse models are selected to associate different dimensions of genomic variation with trait phenotypic variation (G2P models), within the context of the breeding program reference population of genotypes (RPG) and target population of environments (TPE) (Fig. 1; Hammer et al. 2006; Messina et al. 2011; Voss-Fels et al. 2019a). The ensembles of genomic prediction models are designed such that the contributing G2P models can capture different dimensions of trait genetic architecture (Fig. 2; Messina et al. 2025; Tomura et al. 2025a). We illustrate elements of a theoretical framework that can be applied to investigate indicators of the potential benefits for crop breeding applications from these diverse ensembles of G2P prediction models (Fig. 2; Tomura et al. 2025a; Messina et al. 2025).

Page (2010, 2018) introduces the Diversity Prediction Theorem, with examples of approaches for designing ensembles of models for analysis and prediction of properties of a range of complex systems. In summary, the theorem, Eq. (1), states that the error of the ensemble of *i* = *1, …, n* prediction models (*M*) is expected to be less than the average of the errors of the *n* models taken individually, by an amount associated with the diversity of the prediction skill among the *n* individual models (Fig. 1);

1 $$\left( {\widehat{{x^{M} }} - x^{T} } \right)^{2} = \mathop \sum \limits_{i = 1}^{n} \frac{{\left( {x_{i}^{M} - x^{T} } \right)^{2} }}{n} - \mathop \sum \limits_{i = 1}^{n} \frac{{\left( {x_{i}^{M} - \widehat{{x^{M} }}} \right)^{2} }}{n}$$

where ${x}_{i}^{M}$ is the predicted value using the *i*^*th*^ model *M*, ${x}^{T}$ is the true value *T*, and $\widehat{{x}^{M}}$ is the predicted mean calculated over all models used in the ensemble (Figs. 1 and 2). Applying the diversity prediction theorem, Messina et al. (2025) describe a framework for design of model ensembles to exploit different levels of trait G2P dimensionality and enhance prediction of complex traits for breeding applications. They discuss the potential contributions of diverse types of G2P models to both reduce prediction errors and improve prediction accuracy. This framework also highlights the importance of design of breeding METs for creation of effective model ensembles (Fig. 1). Beyond the important considerations of experimental design and numbers of locations and years, there is critical need for careful attention to the representation of the different envirotypes of the TPE within the MET (Cooper et al. 2014a,b, 2023b). Such representation of envirotypes in the MET provides the foundation for design of suitable training data sets that expose genetic variation for the different genetic and physiological dimensions of traits relevant for the TPE (Podlich et al. 1999; Chapman et al. 2003; Messina et al. 2018; Diepenbrock et al. 2022; Cooper and Messina 2023; Washburn et al. 2025; Werner et al. 2025; Tomura et al. 2025a).

With the ongoing advances in computational capacity, the emergence of artificial intelligence and machine learning (AI-ML) technologies with applications to the “big questions” in biology has introduced these new disciplines to the prediction objectives of plant breeding. There is increasing interest in applications of different AI-ML algorithms to support prediction for genetic gain in crop breeding (Hayes et al. 2023; Negus et al. 2024; Washburn et al. 2025; Zhu et al. 2024; Crossa et al. 2025; Messina et al. 2025; Sangjan et al. 2025; Escamilla et al. 2025). However, to date there has been limited attention to designing appropriate experiments to leverage the strengths of AI-ML for the important prediction questions for crop breeding that are related to creating and navigating selection trajectories across breeding cycles (Wisser et al. 2019; Technow et al. 2021; Powell et al. 2022, 2025; Hayes et al. 2023; Choquette et al. 2023; Werner et al. 2025; Polzer et al. 2025). The focus to date has been largely on applying current AI-ML methods to the traditional data sets generated from and adjacent to breeding programs (Fig. 1; Cooper et al. 2014a,b; Rebetzke et al. 2013; Vadez et al. 2015; McCormick et al. 2021; Varshney et al. 2021a,b; Negus et al. 2024; Crossa et al. 2025; Washburn et al. 2025; Sangjan et al. 2025; Escamilla et al. 2025).

While access to AI-ML has stimulated the recent interest in prediction for breeding, it is important to recognize that the ambition to use prediction methods to enhance the effectiveness of plant breeding programs is not new. There is a long history of successful development of measurement technologies and associated G2P modelling methodology to support prediction for crop breeding applications (Hanson and Robinson 1963; Henderson 1975; Meuwissen et al. 2001; Holland et al. 2002; Hammer et al. 2006; Boer et al. 2007; Bernardo and Yu 2007; Bernardo 2008, 2016, 2020; Moose and Mumm 2008; van Eeuwijk et al. 2010; Cooper et al. 2014a,b; Voss-Fels et al. 2019a; Cobb et al. 2019; Washburn et al. 2025; Crossa et al. 2025). We can build on the key lessons from this history and the foundations that have been established from these historical efforts, to focus and enhance experiment design for future investigations into the potential of AI-ML technologies for breeding (Messina et al. 2025; Sangjan et al. 2025; Escamilla et al. 2025).

Following the framework of von Rueden et al. (2023), we review some relevant background to highlight important connections between prior knowledge of trait genetic architecture and the different prediction methods of the past and the emerging AI-ML applications. We emphasise opportunities to incorporate prior knowledge of trait genetic architecture and design experiments to leverage the strengths of AI-ML for breeding applications (Fig. 1; Cooper and Messina 2023; Negus et al. 2024; Messina et al. 2025; Crossa et al. 2025; Powell et al. 2025; Sangjan et al. 2025; Escamilla et al. 2025).

While investigating the opportunities for any technology, including AI-ML for prediction, it is important to recognise all technologies used in breeding have and will continue to go through a life cycle that includes discovery, development, refinement, and for some technologies mature application stages (Campos et al. 2004; Bernardo 2016, 2020; Escamilla et al. 2025). Some have made important and lasting contributions, e.g., mixed model methodology and Best Linear Unbiased Prediction (Henderson 1975; Gilmour et al. 1997; Smith et al. 2001; Boer et al. 2007; Bernardo and Yu 2007; van Eeuwijk et al. 2010). Others have had a shorter life cycle and made more limited direct contributions, e.g., marker-assisted selection for yield improvement based on large-effect quantitative trait loci (QTL) (Bernardo 2008, 2016, 2020). Other technologies have had a longer life cycle with less direct impact on yield improvement and genetic gain, e.g., transgenics for yield (Campos et al. 2004; Barker et al. 2005; Guo et al. 2014; Simmons et al. 2021; Khaipho-Burch et al. 2023; Linares et al. 2023, 2024, 2025). While the direct contributions of transgenics to genetic gain for yield have been modest to date, many investigations employing transgenic technologies have enabled important discoveries that continue to help shape our current understanding of trait genetic architecture (e.g., Fig. 2; Dong et al. 2012). Collectively these technologies and the associated discoveries inform and provide the foundations for the current progress in developing operational prediction-based crop breeding methodology (Bernardo 2008, 2016, 2020; Cooper et al. 2014a,b; Crossa et al. 2014; Voss-Fels et al. 2019a; Werner et al. 2025; Escamilla et al. 2025).

We argue that we are currently in the discovery and development phases of the AI-ML life cycle for breeding applications (Bernardo 2016, 2020). The results reported from the current range of AI-ML applications to breeding provide valuable insights into the strengths and weaknesses of these technologies and continue to identify new directions to refine and apply AI-ML methods and design experiments to leverage their strengths (Technow et al. 2015, 2021; Powell et al. 2022, 2025; Hayes et al. 2023; Sangjan et al. 2025; Escamilla et al. 2025). Messina et al. (2025) introduce a theoretical framework for G2P prediction that can be applied to evaluate the current and investigate potential new contributions of AI-ML for predictive breeding applications.

Applying the framework of Messina et al. (2025), we emphasise key aspects of the “breeding context” throughout this review. A common challenge for all trait mapping and prediction investigations is the difficulty of projecting the results from the limited sampling of the G2P dimensionality observable within the discovery research context of a MET, to the high G2P dimensionality encountered within the diversity of Genotype-by-Environment-by-Management (GxExM) contexts of the TPE (Fig. 1; Ceccarelli 1989, 1994; Tardieu 2012; Peng et al. 2020; Ceccarelli and Grando 2020; Bernardo 2020; Rogers et al. 2021; Langridge et al. 2021; Kholová et al. 2021; Cooper et al. 2020, 2023a,b; Lopez-Cruz et al. 2023; Escamilla et al. 2025). The need to adequately represent the reference population of genotypes (RPG) and the target population of environments (TPE) is a recurring challenge for the design of multi-environment trials (METs) at all stages of breeding programs, and also for the design of specialised trait mapping studies and for applications of AI-ML technologies (Atlin et al. 2001; Boer et al. 2007; Gaffney et al. 2015; Gage et al. 2020; Cooper et al. 2014a,b; 2023a,b; Werner et al. 2025; Escamilla et al. 2025). Throughout this review we will highlight areas where “breeding context” matters in the design of METs and adjacent experiments in specialised facilities that are used to generate the data to train G2P models for prediction-based breeding applications (Fig. 1; Messina et al. 2025; Sangjan et al. 2025; Escamilla et al. 2025; Powell et al. 2025). This is recognised as a general issue for successful “out of sample” prediction (van Eeuwijk et al. 2016; Cooper et al. 2020; von Rueden et al. 2023; Messina et al. 2025).

Interest continues to grow in the design of integrated applications of genomics (Fig. 1; Edwards and Batley 2010; Yuan et al. 2017; Bayer et al. 2020), phenomics (Fig. 1; Araus and Cairns 2014; Araus et al. 2018; Reynolds et al. 2020; Smith et al. 2021), and enviromics (Fig. 1; Chapman et al. 2000; Löffler et al. 2005; Chenu et al. 2011; Xu 2016; Cooper et al. 2020; Costa-Neto et al. 2021a,b; Lopez-Cruz et al. 2023) within breeding experiments to provide the “big data” required to leverage the strengths of AI-ML modelling technologies (e.g., Kusmec et al. 2021; Varshney et al. 2021a,b; Zhu et al. 2024; Crossa et al. 2025; Washburn et al. 2025; Sangjan et al. 2025). From a plant breeding perspective, seeking an improved understanding of trait genetic architecture through such applications of AI-ML technologies is motivated by creating new opportunities to use the constructed trait G2P models to enhance breeding methodology, accelerate rates of genetic gain and design novel breeding outcomes (Voss-Fels et al. 2019a; Hayes et al. 2023; Escamilla et al. 2025).

To address the call to action for integration of technologies for crop breeding, we consider four interrelated themes: (1) First, we consider the definition of trait genetic architecture in the genomics era from the perspective of a plant breeding context; specifically considering breeding objectives and target product profiles, the standing genetic variation for traits in the RPG, and the GxExM context of the TPE (Bernardo 2020; Kholová et al. 2021; Technow et al. 2021; Cooper et al. 2021a,b, 2023a,b). (2) Second, we provide an historical overview of lessons from some key technologies and research methods that have been developed to enhance prediction capabilities for crop breeding applications: crop physiology to define ideotypes as breeding targets, QTL mapping and marker-assisted selection (MAS) and transgenics for yield improvement (Jackson et al. 1996; Campos et al. 2004; Hammer et al. 2006). (3) Third, we consider the potential to improve crop growth models (CGM) in combination with G2P models of trait genetic architecture as a CGM-G2P framework to enhance the use of prior trait knowledge for prediction-based breeding applications (Chapman et al. 2003; Messina et al. 2009, 2011; Cooper et al. 2014a,b, 2021b; Bustos-Korts et al. 2019; Powell et al. 2025; Escamilla et al. 2025). (4) Fourth, we apply the framework of Messina et al. (2025) to consider the contributions of the diverse ensembles of G2P models from quantitative genetics and AI-ML for breeding-focused prediction applications (Bian and Holland 2015; McCormick et al. 2021; Kick and Washburn 2023; Tomura et al. 2025a; Kawakita et al. 2024; Washburn et al. 2025). Within each theme we introduce relevant background and consider experiences gained from applications to commercial maize breeding (Duvick 2001; Duvick et al. 2004; Campos et al. 2004; Barker et al. 2005; Cooper et al. 2014a,b; Gaffney et al. 2015; McFadden et al. 2019; Simmons et al. 2021; Linares 2023, 2024, 2025; Messina et al. 2023a,b; Cooper and Messina 2023; Rotundo et al. 2025). We highlight key lessons that we anticipate will have general relevance for prediction-based breeding for all crops.

Collectively these four themes provide background and motivations to investigate ensembles of G2P models that combine the prediction capabilities of conventional quantitative genetics (e.g., Meuwissen et al. 2001; Bernardo and Yu 2007) and AI-ML technologies (e.g., Negus et al. 2024; Crossa et al. 2025) in combination with methods that utilise our prior understanding of trait genetic architecture, including CGM-G2P (e.g., Technow et al. 2015; Messina et al. 2018; Diepenbrock et al. 2022; Kawakita et al. 2024). We conclude by discussing some promising emerging future directions for prediction-based crop breeding.

### Theme 1: trait genetic architecture and standing genetic variation for breeding

Following the definitions provided by Mackay (2001), Holland (2007), Cooper et al. (2005, 2009), Marjoram et al. (2014), and Boyle et al. (2017), Table 1 summarises key components of trait genetic architecture. As high-throughput measurement technologies and G2P modelling capabilities have advanced, applications to suitable data sets have revealed new G2P dimensions of trait genetic architecture (Buckler et al. 2009; Messina et al. 2018; Wisser et al. 2019; Varshney et al. 2021b; Diepenbrock et al. 2022; Washburn et al. 2025; Tomura et al. 2025a). Elements of the infinitesimal model, such as large numbers of influential regions distributed throughout the genome, are still applicable (Falconer and Mackay 1996; Lynch and Walsh 1998; Walsh and Lynch 2018; Bernardo 2020). However, new details of the diverse roles of sequence variation at these many regions, and the influences of the networks of intermediate biological layers that create the G2P dimensionality between sequence and trait phenotypes, challenge the assumption of consistent effects of alleles and allele substitution effects within and among the populations of genotypes developed by breeding programs (Cheverud and Routman 1995; Cooper et al. 2005; Hammer et al. 2006; Marjoram et al. 2014; Guo et al. 2014; Boyle et al. 2017; Ramstein et al. 2019; Wisser et al. 2019; Technow et al. 2021; Powell et al. 2021, 2022; Diepenbrock et al. 2022; Linares et al. 2023, 2024, 2025; Escamilla et al. 2025).

**Table 1:** Plant breeding perspective of components of trait genetic architecture and some considerations for genome to phenome (G2P) modelling for prediction and selection within the reference population of genotypes (RPG) for a target population of environments (TPE) (Fig. 1)

Component	Considerations	References
QTL number	Effective population size in terms of founder genotypes and structure of the breeding program RPG Cross designs and the genotype and environment sample sizes used for trait mapping Genome distribution of realised levels of recombination and resulting patterns of linkage disequilibrium Prior knowledge of candidate genes influencing target traits and their positions within founder haplotypes Emergence of new QTL from sources including mutations and release of constraints acting on cryptic variation within the RPG Addition of new QTL through introgression of new sources of genetic diversity into the RPG	Schon et al. (2004), Campos et al. (2004), Salvi et al. (2007), Buckler et al. (2009), Durand et al. (2010), van Eeuwijk et al. (2010), Guo et al. (2014), Cooper et al. (2014a,b), Wisser et al. (2019), Chen et al. (2019), Bernardo 2020), Simmons et al. (2021)
QTL alleles	Number of alleles and their frequencies within the RPG Mapping of QTL alleles to founder haplotypes. Structural organization of sequence variation into founder haplotypes, including the arrangement of QTL alleles within founder haplotypes Emergence and creation of novel alleles through sequence manipulation, including mutation, transgenes, editing and synthetic biology design technologies	Campos et al. (2004), Cooper et al. (2014a), Guo et al. (2014), Voss-Fels et al. (2019b), Wurtzel et al. (2019), Jensen et al. (2020), Bayer et al. (2020), Simmons et al. (2021), Linares et al. (2023, 2024, 2025)
QTL effects	Distribution of sizes of allele effects on standing variation for trait phenotypes within the RPG Mechanistic factors that impact the distribution of QTL effect size and their interpretation in terms of mode of gene action	Buckler et al. (2009), Guo et al. (2014), Wisser et al. (2019), Bernardo 2020), Simmons et al. (2021)
QTL modes of action	Methods to quantify allele effects and effects of allele substitution within the RPG Models to distinguish additive and non-additive gene action Breeding context: Defined as the influence of breeding methodology on the structure of the RPG and the evolution of additive and non-additive gene action and genetic variance Within QTL: Additive action is the linear cumulative effect of additional copies of alleles Within QTL: Non-additive dominance gene action is a deviation from the linear cumulative effect of alleles Among QTL: Additive action is the cumulative effect across the individual within QTL effects. The breeding value of an individual is determined by the summation of the within QTL additive effects across the multiple QTL contributing to the traits Among QTL: Non-additivity is attributed to deviations from the expected summation of additive effects across the QTL. Statistical non-additive effects across QTL are attributed to interactions among the QTL and often considered to be a consequence of biological epistasis Among QTL Cis-Action: Occurs when different regions of contiguous sequence interact to influence the effect of a QTL on a trait phenotype. For example, Cis-Action can result from variation in the promoter sequence regulating differential expression of the coding sequence of a gene, contributing to variation for timing and levels of gene expression and trait effects Among QTL Trans-Action: Occurs when sequence from non-contiguous regions of a genome interact to influence the effect of a QTL on a trait phenotype. For example, Trans-Action can result from variation in transcription factors that bind to promoters of genes to influence timing and levels of gene expression	Cheverud and Routman 1995), Falconer and Mackay 1996), Lynch and Walsh 1998), Duvick 2001), Duvick et al. (2004), Cooper et al. (2005), Salvi et al. (2007), van Eeuwijk et al. (2010), Guo et al. (2014), Dong et al. (2012), Huang and Mackay 2016), Boyle et al. (2017), Walsh and Lynch 2018), Bernardo 2020), Technow et al. (2021), Simmons et al. (2021)
QTL by Environment Interactions	Magnitude and frequency of QTL by environment interactions within the RPG and TPE breeding context As with genotype by environment interactions, distinguish between heterogeneity of variance scale across environments and cross-over interactions that change the ranking of the allele effects on trait phenotypes across the envirotypes within the TPE and the diversity of environments within envirotypes Differences in numbers and effects of QTL for the different envirotypes of the TPE Emergence of new QTL with changes in the envirotype composition of the TPE due to consequences of climate change, new agronomic management practices, new and increased occurrences of biotic stresses	Campos et al. (2004), Boer et al. (2007), Cooper et al. (2009, 2014a,b), Messina et al. (2011, 2018), Guo et al. (2014), van Eeuwijk et al. (2016), Millet et al. (2019), Bustos-Korts et al. (2019), Desbiez-Piat et al. (2023), Choquette et al. (2023)

New opportunities are continually emerging to investigate the details of trait genetic architecture and develop novel G2P models that can be utilised to improve prediction for breeding applications (Hammer et al. 2006; Marjoram et al. 2014; Technow et al. 2015; Wang et al. 2023; Messina et al. 2025; Tomura et al. 2025a; He et al. 2025). We consider how to include prior knowledge of the roles of multiple QTL within networks that operate to determine trait phenotypes. The networks can be defined within and across different levels of the biological hierarchy from genome sequence to trait phenotype (Cooper et al. 2005, 2021b; Hammer et al. 2006; Messina et al. 2009; Marjoram et al. 2014; Dong et al. 2012; Marshall-Colon et al. 2017; Boyle et al. 2017; Powell et al. 2025). This includes consideration of the roles of gene regulatory networks and related mechanistic molecular networks, and trait networks operating at the plant organ, whole-plant and crop levels (Messina et al. 2009, 2011; Dong et al. 2012; Tardieu et al. 2021; Gleason et al. 2022; Powell et al. 2022). Crop Growth Models provide an integrative CGM-G2P framework for studying trait networks operating at different levels of the genome to trait phenotype hierarchy (Cooper et al. 2002, 2021b; Messina et al. 2009, 2018; Technow et al. 2015; Chenu et al. 2017; Hammer et al. 2019;).

Plant breeders study trait genetic architecture (Table 1) to seek new opportunities to accelerate rates of genetic gain and delivery of on-farm impact achieved through deployment and adoption of improved products (Fig. 1; Campos et al. 2004; Barker et al. 2005; Cooper et al. 2014a,b; Gaffney et al. 2015; McFadden et al. 2019; Voss-Fels et al. 2019a,b; Bernardo 2020; Kholová et al. 2021; Messina et al. 2023a,b; Werner et al. 2025). Whole genome prediction (WGP) has emerged as the preferred foundation for the redesign of crop breeding methodology to leverage genome sequence information and accelerate genetic gain for quantitative traits (Meuwissen et al. 2001; Bernardo and Yu 2007; Heffner et al. 2009; Cooper et al. 2014a,b; Voss-Fels et al. 2019a,b; Bernardo 2020; Varshney et al. 2021b; Khaipho-Burch et al. 2023; Crossa et al. 2025; Escamilla et al. 2025). However, there is still much to be done to fully leverage the potential of WGP to increase the efficiency and scale of breeding programs (Voss-Fels et al. 2019a,b; Messina et al. 2022, 2025; Mascher et al. 2024; Sangjan et al. 2025; Escamilla et al. 2025; Powell et al. 2025). We are motivated to extend the definition of trait genetic architecture (Table 1) to include explicit consideration of the organisation of QTL interactions within networks to highlight a potential target for applications of AI-ML technologies for G2P prediction (e.g., Podlich et al. 2004; Cooper et al. 2005; Hammer et al. 2006; Dong et al. 2012; Marjoram et al. 2014; Huang and Mackay 2016; Boyle et al. 2017; Technow et al. 2021; Powell et al. 2022, 2025; Tomura et al. 2025a; Messina et al. 2025).

The statistical G2P models of quantitative genetics, that have predominantly been used to date to enable WGP, are effective (Meuwissen et al. 2001; Cooper et al. 2014a,b; Voss-Fels et al. 2019a; Escamilla et al. 2025). However, they do not fully leverage our continually growing body of prior knowledge of the genetic and physiological architecture of quantitative traits (Hammer et al. 2006; Messina et al. 2009, 2025; Marjoram et al. 2014; Huang and Mackay 2016; Boyle et al. 2017; Cooper et al. 2021b; Bernardo 2020). Comparisons with WGP results achieved from applications of AI-ML algorithms have demonstrated that the AI-ML algorithms can also be competitive with the statistical models (Wang et al. 2023; Crossa et al. 2025; Barreto et al. 2024; Tomura et al. 2025a; He et al. 2025). However, the general finding is that the preferred G2P model for prediction is context dependent across MET data sets, traits and crop targets. This is in accordance with the general expectations of the “No Free Lunch (NFL) Theorem” (Wolpert and Macready 1997). What does this mean for WGP applications to crop breeding? First, we propose an important implication of the NFL theorem; that across high dimensional G2P problems with a diversity of complex state spaces (Cooper et al. 2005; Messina et al. 2011; Marjoram et al. 2014; Boyle et al. 2017; Technow et al. 2021), no one WGP algorithm will be superior on average across all trait-crop G2P state spaces. Second, if we accept the body of empirical results and the implication of the NFL theorem, then to tackle the ubiquitous context dependency of G2P dimensionality we should explore and leverage the diversity of results from the alternative prediction algorithms (Fig. 1; Messina et al. 2025). A first step in the path forward is to consider the opportunities to design ensembles of prediction algorithms and to enable ensemble-breeding that leverages networks of breeding programs (Cooper et al. 2014a; Garrett et al. 2017; Technow et al. 2021; Tomura et al. 2025a; Messina et al. 2025).

Applying the framework of Messina et al. (2025) we review emerging opportunities to advance WGP models in ways that enable them to more effectively leverage prior biological knowledge of QTL and traits operating in hierarchical G2P trait networks. To clarify the opportunities to utilise such prior knowledge for prediction, we recognise classes of G2P models as “symbolic” and “sub-symbolic”, as defined by von Rueden et al. (2023) and Messina et al. (2025). Symbolic models are based on algorithms that explicitly use prior knowledge to constrain the G2P solution space by defining biophysical limits: e.g., use of crop growth models (Hammer et al. 2006, 2019; Messina et al. 2009; Technow et al. 2015), and genetic regulatory networks such as those developed for flowering time prediction in maize (Dong et al. 2012). These symbolic models are where much of our prior knowledge of trait biology resides (Hammer et al. 2006, 2019; Messina et al. 2025). Sub-symbolic models are based on algorithms that use other forms of regularization (e.g., Pérez and de los Campos 2014), inductive inference (Mitchell 2020) and various forms of AI-ML (Ersoz et al. 2020; Washburn et al. 2021; Colantonio et al. 2022; Wang et al. 2023; Negus et al. 2024; Crossa et al. 2025; He et al. 2025). New understanding of trait G2P architecture may arise from this sub-symbolic domain and be captured through improvements to the symbolic models (Messina et al. 2025). Hybrid models that combine elements of both approaches are referred to as fusion models (e.g., Messina et al. 2009, 2018, 2025; Technow et al. 2015; Diepenbrock et al. 2022; Jighly et al. 2023a,b; von Rueden et al. 2023). The motivation for considering these alternative G2P modelling approaches for WGP is to review opportunities to more effectively utilise prior biological knowledge to inform WGP as we redesign breeding programs to create new opportunities for crop improvement and accelerate genetic gain (Hammer et al. 2006; Cooper et al. 2014a,b, 2021a; Voss-Fels et al. 2019a; Simmons et al. 2021; Cooper and Messina 2023; Hayes et al. 2023; von Rueden et al. 2023; Khaipho-Burch et al. 2023; Polzer et al. 2025; Escamilla et al. 2025). One clear target is to accelerate the development of new genotypes with improved climate resilience to support and enable sustainable and regenerative agricultural practices (Brummer et al. 2011; Chapman et al. 2012; Langridge et al. 2021; Kholová et al. 2021; Messina et al. 2022, 2023a,b; Moore et al. 2023; Pixley et al. 2023; Cooper and Messina 2023; Escamilla et al. 2025).

### Theme 2: sources of prior information to predict trait phenotypes

Beyond the classical prediction framework of the breeder’s equation from quantitative genetics (Lush 1937; Hanson and Robinson 1963; Falconer and Mackay 1996; Lynch and Walsh 1998; Holland et al. 2002; Moose and Mumm 2008; Walsh and Lynch 2018; Bernardo 2020; Escamilla et al. 2025), other approaches, that utilise model-based capabilities, have been investigated for their potential to accelerate breeding outcomes (Hammer et al. 2006; Cooper et al. 2014a; Voss-Fels et al. 2019a; Escamilla et al. 2025). Here we highlight three areas that have been considered to enable enhanced crop yield prediction: (1) crop physiology and ideotype breeding, (2) QTL mapping and marker-assisted selection, (3) transgenics for yield improvement. Results and experience from applications of these domains to maize breeding provide important insights into our current understanding of trait genetic architecture (Table 1) and can guide potential applications of AI-ML for crop breeding applications (Cooper et al. 2014a; Hayes et al. 2023; Messina et al. 2023a, 2025; Cooper and Messina 2023; Escamilla et al. 2025).

#### Theme 2.1: breeding lessons from crop physiology and crop ideotypes

Plant and crop physiology have a long history of investigating traits that contribute to genotype adaptation and the performance differences among genotypes for biomass and grain yield and other complex traits (Donald 1968; Passioura 1977; Monteith 1977; Ludlow and Muchow 1990; Jackson et al. 1996; Cooper and Hammer 1996; Richards et al. 2002; Hammer et al. 2006; Richards 2006; Messina et al. 2009, 2011; Fischer et al. 2014; Reynolds and Langridge 2016; Araus et al. 2018; Tardieu et al. 2021; Welcker et al. 2022). Yield outcomes at the crop level have been of primary interest as targets for physiology informed predictions applied to breeding (Cooper and Hammer 1996; Fischer et al. 2014). Detailed physiological investigations have often been limited to small numbers of genotypes. With availability of high-throughput phenomics and enviromics technologies, investigations have been extended to larger numbers of genotypes to enable investigation of trait heritability and genetic architecture at scales relevant for the breeding context (Fig. 1; Richards 2006; Chenu et al. 2011; Rebetzke et al. 2013; Araus and Cairns 2014; Cooper et al. 2014a,b; Vadez et al. 2015; Reynolds and Langridge 2016; Araus et al. 2018; Reynolds et al. 2020; Smith et al. 2021; Zhao et al. 2022; Welcker et al. 2022; Cooper and Messina 2023).

Physiological frameworks that include prior knowledge of phenological development, biomass accumulation and partitioning have been proposed to support crop yield prediction (Passioura 1977; Monteith 1977; Cooper and Hammer 1996; Richards et al. 2002; Richards 2006; Messina et al. 2009, 2011; Fischer et al. 2014; Reynolds and Langridge 2016; Chenu et al. 2017; Hammer et al. 2019). Applying these frameworks, individual traits and networks of traits have been investigated for their roles in yield determination (Ludlow and Muchow 1990; Richards et al. 2002; Hammer et al. 2006; Messina et al. 2009, 2011; Chenu et al. 2017; Zhao et al. 2022; Gleason et al. 2022; Welcker et al. 2022; Cooper and Messina 2023). Ideotypes, based on preferred combinations of traits, have been defined as targets to realise the yield objectives of breeding programs (Donald 1968; Richards et al. 2002; Richards 2006; Hammer et al. 2009; Messina et al. 2011; Reynolds and Langridge 2016; Gleason et al. 2022; Zhao et al. 2022; Pixley et al. 2023). These diverse models of the prior knowledge of physiological determinants of yield are fundamentally hierarchical. Collectively we refer to these as crop growth models (CGMs). In some applications, the CGM has been extended to include trait genetics (White and Hoogenboom 1996, 2003; Cooper et al. 2002, 2021b; Chapman et al. 2003; Hammer et al. 2006; Messina et al. 2006, 2009, 2011, 2018; Chenu et al. 2009; Dong et al. 2012; Technow et al. 2015; Bustos-Korts et al. 2019; Diepenbrock et al. 2022; Zhao et al. 2022).

Within the framework of informed AI-ML (von Reuden et al. 2023), CGMs use combinations of symbolic relationships to quantify prior knowledge of the mechanistic interactions between traits and environmental conditions that determine higher level trait outcomes of genotypes at the crop grain yield level for diverse agronomic management and environmental conditions (Cooper et al. 2002; Chapman et al. 2003; Hammer et al. 2006, 2019; Messina et al. 2009; Holzworth et al. 2014; Technow et al. 2015; Jones et al. 2016; Chenu et al. 2017; Peng et al. 2020; Gleason et al. 2022).

Many plant and crop physiology investigations applying CGMs sought traits that operated at lower levels in the hierarchical framework, that could be more easily measured than grain yield and were predictive of the yield variation among genotypes (Cooper et al. 2002; Hammer et al. 2006; Richards 2006; Messina et al. 2009; Reynolds and Langridge 2016; Tardieu et al. 2021; Welcker et al. 2022). These physiology-inspired investigations have been valuable sources of information to understand the roles of different traits and trait combinations in determining the crop yield performance of genotypes in the TPE of breeding programs (Campos et al. 2004; Cooper et al. 2014a,b; Gaffney et al. 2015; Zhao et al. 2022; Welcker et al. 2022; Messina et al. 2023a,b). However, to date, the direct contributions of the ideotype approaches for predictive breeding applications have been limited in comparison to the successful outcomes of breeding for the end-point trait yield (Fig. 1; Campos et al. 2004; Duvick et al. 2004; Richards 2006; Cooper and Messina 2023).

Three key lessons for prediction-based breeding:

1. Breeding context of the RPG and TPE is important for any investigation of trait genetic architecture (Table 1; Richards et al. 2002; Richards 2006; Messina et al. 2009; Tardieu 2012; Cooper et al. 2014a; Reynolds and Langridge 2016; Bernardo 2020; Tardieu et al. 2021; Escamilla et al. 2025). Individual traits that contribute to the yield of genotypes can rarely be studied in isolation from context of design of selection strategies to accelerate breeding outcomes at the level of crop yield (Duvick et al. 2004; Campos et al. 2004; Hammer et al. 2009; Messina et al 2023a). It follows that the genetic architecture of traits, investigated applying plant and crop physiological frameworks, is expected to be context dependent (Richards 2006; Messina et al. 2009, 2011, 2023a; Reynolds and Langridge 2016; Cooper and Messina 2023).
2. The successful crop ideotypes identified from physiology investigations conducted to improve our understanding of crop yield can inform breeding directions (Richards et al. 2002; Richards 2006; Hammer et al. 2009; Reyes et al. 2015; Zhao et al. 2022; Cooper and Messina 2023). However, crop yield improvement has largely progressed by selection directly on yield rather than via selection for crop ideotypes (Campos et al. 2004; Duvick et al. 2004; Richards 2006; Messina et al. 2011; Cooper et al. 2014a,b; Gaffney et al. 2015; Cooper and Messina 2023).
3. Crop Growth Models (CGMs), combined with high-throughput phenomics and enviromics, can be used to scale physiology investigations to broaden their applications to the “breeding context” and use prior knowledge of traits and trait combinations for applications within breeding programs (Cooper et al. 2002; Hammer et al. 2006; Messina et al. 2009, 2011, 2023a; Zhao et al. 2022; Cooper and Messina 2023; Amin et al. 2025; Escamilla et al. 2025).

#### Theme 2.2: breeding lessons from QTL mapping and marker-assisted selection

The first-generation of molecular marker technologies provided an entry point to investigate the genetic architecture of traits (Table 1; Patterson et al. 1988, 1991; Campos et al. 2004; Dudley 2007; Bernardo 2008, 2016, 2020). The early discoveries of modest numbers of QTL and estimates of relatively large effects for a small number of QTL, created optimism for targeted marker-assisted selection (MAS), based on the large-effect QTL for quantitative traits. However, except in a few successful applications to specific traits, most of these early results from QTL studies did not translate into the anticipated widespread acceleration of genetic gain for yield and other complex traits for the breeding context (Lande and Thompson 1990; Barker et al. 2005; Eathington et al. 2007; Moose and Mumm 2008; Cobb et al. 2018, 2019; Bernardo 2008, 2016, 2020). Since the early QTL mapping studies, experimental investigations, and considerations from statistical sampling theory, have clarified the need to work with large genotype and environment samples, based on 100s of crosses and 1000s of individuals relevant to the breeding program. These large samples are required to reliably represent genetic diversity within the RPG and the environmental diversity of the TPE of the breeding program (Beavis et al. 1994; Melchinger et al. 1998; Schon et al. 2004; Campos et al. 2004; Podlich et al. 2004; Boer et al. 2007; Bernardo 2008; Cooper et al. 2014a,b; Millet et al. 2019).

Multi-parental designs and association mapping, based on large samples of genotypes with known genetic relationships, were proposed as approaches to overcome some of the limitations of the early biparental mapping studies. Nested association mapping (NAM) designs that combine elements of association mapping and segregation analysis within populations were developed and applied to study the genetic architecture of traits (Yu et al. 2006; Buckler et al. 2009; Chen et al. 2019; Gage et al. 2020). While these approaches improved mapping resolution, the resulting information on trait genetic architecture (Table 1) was often distinct from the standing genetic variation of breeding programs, limiting direct applications for prediction-based breeding (Bernardo 2008, 2016, 2020; Cooper et al. 2014a,b; Gage et al. 2020). Many of the early lessons from attempts to apply results from QTL mapping studies to maize breeding, guided strategies for developing appropriate training populations for successful future applications of WGP (Podlich et al. 2004; Campos et al. 2004; Barker et al. 2005; Cooper et al. 2014a,b; Gaffney et al. 2015; Bernardo 2008, 2016, 2020; Cobb et al. 2018, 2019; Voss-Fels et al. 2019a; Messina et al. 2018, 2023a,b; Diepenbrock et al. 2022; Cooper and Messina 2023; Polzer et al. 2025; Escamilla et al. 2025).

Beyond the end point complex traits targeted by breeding programs, investigations have been undertaken to use results obtained from QTL mapping of specific traits in combination with crop physiological frameworks and CGMs to improve prediction of genotype yield performance for target environments (White and Hoogenboom 1996, 2003; Chapman et al. 2003; Campos et al. 2004; Barker et al. 2005; Yin et al. 2005; Cooper et al. 2002, 2005; Hammer et al. 2006; Messina et al. 2006, 2011; Sadok et al. 2007; Tardieu 2012; Tardieu et al. 2021). Strategies for using CGMs to enhance WGP emerged (Hammer et al. 2006; Cooper et al. 2014a,b, 2021b; Technow et al. 2015; Onogi et al. 2016; Messina et al. 2018; Toda et al. 2020; Diepenbrock et al. 2022; Jighly et al. 2023a,b; Cooper and Messina 2023; Escamilla et al. 2025). Using specific trait models and the CGM as an integrated G2P link function within WGP methods, the CGM can be used in combination with appropriate training data sets to evaluate multiple trait combinations for yield prediction skill. With appropriately designed training data sets, applications of the CGM-WGP methodology enables millions of ideotypes to be evaluated within the WGP framework and for the context of the RPG (Fig. 1; Cooper et al. 2014a,b; Technow et al. 2015; Messina et al. 2018; Diepenbrock et al. 2022; Cooper and Messina 2023). Further, the predictive skill of the CGM-WGP methodologies can be compared directly with any other prediction methods and its contributions to ensemble-based prediction can be optimised as part of the WGP framework (Fig. 1; Kawatika et al. 2024; Messina et al. 2025; Powell et al. 2025).

Three key lessons for prediction-based breeding:

1. Breeding context is important for investigation of trait genetic architecture through QTL mapping (Table 1). QTL operate within networks of background genetic variation. Their effects on traits and yield vary with the RPG and the TPE (Fig. 1; Cooper et al. 2005, 2009; Tardieu 2012; Bernardo 2008, 2016, 2020; Escamilla et al. 2025).
2. Yield related traits that are targeted for improvement in breeding programs are under the control of large numbers of QTL, most of which make small contributions to the standing genetic variation in the RPG for the TPE (Schon et al. 2004; Boer et al. 2007; Bernardo 2020; Cooper et al. 2014a,b; Millet et al. 2019; Polzer et al. 2025).
3. Marker-assisted selection based on small numbers of specific QTL for yield improvement is possible through understanding how the traits contribute to yield in specific environments (Richards 2006; Reynolds and Langridge 2016; Cobb et al. 2018; Bernardo 2020; Zhao et al. 2022). However, as for physiology investigations based on limited sets of traits, marker-assisted selection based on small numbers of large effect QTL has had limited impact on genetic gain for yield in comparison to direct selection on yield in the TPE (Campos et al. 2004; Barker et al. 2005).

#### Theme 2.3: breeding lessons from transgenic approaches applied to yield and complex traits

In addition to QTL mapping, transgenic methods have been used to study trait genetic architecture and validate QTL identified through mapping (Campos et al. 2004; Salvi et al. 2007; Meng et al. 2011; Guo et al. 2014; Stephenson et al. 2019; Simmons et al. 2021; Linares et al. 2023, 2024, 2025). Transgenic approaches have been used successfully to add important novel sources of genetic diversity for resistance to insect pests of crops and to provide tolerance to herbicides to augment weed control strategies in cropping systems. However, these same discovery methods when applied to complex traits such as yield and related agronomic traits have had less impact on genetic gain for the TPE (Campos et al. 2004; Barker et al. 2005; Simmons et al. 2021; Khaipho-Burch et al. 2023; Linares et al. 2023, 2024, 2025). The challenges encountered in translating the many important gene discoveries into prediction-based breeding applications have provided valuable insights into the context-dependent nature of individual gene contributions to trait phenotype performance for the large numbers of genotypes created by breeding programs (Campos et al. 2004; Guo et al. 2014; Cooper et al. 2014a; Simmons et al. 2021; Khaipho-Burch et al. 2023; Linares et al. 2023, 2024, 2025). The many potential interactions between the genes targeted for manipulation by transgenic approaches and the standing genetic variation for other genes involved in the trait genetic architecture within the RPG, highlight the potential influences of gene networks on sources of non-additivity for G2P prediction and breeding to improve yield for a TPE (Campos et al. 2004; Cooper et al. 2005; Hammer et al. 2006; Dong et al. 2012; Guo et al. 2014; Technow et al. 2021; Simmons et al. 2021; Linares et al. 2023, 2024, 2025; Escamilla et al. 2025).

Three key lessons for prediction-based breeding:

1. Breeding context for gene discovery and transgene evaluation is important. Individual genes can be identified to have an impact on trait and yield phenotypes within a specific context. However, the transgene effects identified within the discovery context will rarely directly translate into comparable effects for yield within the RPG and TPE of breeding programs (Campos et al. 2004; Simmons et al. 2021; Linares et al. 2023, 2024, 2025).
2. Targeted manipulation of individual genes to create novel alleles that outperform the alleles that contribute to the standing genetic variation for yield related traits in the RPG in breeding programs is difficult (Campos et al. 2004; Guo et al. 2014; Simmons et al. 2021; Khaipho-Burch et al. 2023; Linares et al. 2023, 2024, 2025).
3. Large scale discovery efforts connected to the context of breeding programs are necessary to create novel genetic diversity that can be used within breeding programs (Campos et al. 2004; Simmons et al. 2021; Linares et al. 2023, 2024, 2025).

### Theme 3: CGM-G2P framework for breeding applications

Based on the accumulation of prior knowledge of trait genetic architecture (Table 1; Hammer et al. 2006) from combinations of physiological investigations (Richards et al. 2002; Campos et al. 2004; Richards 2006; Hammer et al. 2009; Reyes et al. 2015; Messina et al. 2009, 2015, 2019, 2021; Tardieu 2012; Reynolds and Langridge 2016), QTL mapping (Campos et al. 2004; Boer et al. 2007; Sadock et al. 2007; Buckler et al. 2009; Tardieu 2012; Chen et al. 2019; Millet et al. 2019; Mayer et al. 2020) and transgenic approaches (Campos et al. 2004; Guo et al. 2014; Simmons et al. 2021; Linares et al. 2023, 2024, 2025), hierarchical G2P maps for traits can be created and investigated at different levels of genomic and phenotypic resolution using CGMs (Cooper et al. 2002, 2021b; Campos et al. 2004; Hammer et al. 2006, 2019; Messina et al. 2009, 2011; Dong et al. 2012; Marshall-Colon et al. 2017; Peng et al. 2020; Powell et al. 2025; Escamilla et al. 2025).

The framework of the CGM can contain multiple layers, with each layer representing the interacting networks of molecular, tissue, organ and whole plant properties of traits (Richards et al. 2002; Cooper et al. 2002, 2005, 2021b; Richards 2006; Sadock et al. 2007; Messina et al. 2009, 2011, 2015, 2025; Hammer et al. 2006, 2019; Reynolds and Langridge 2016; Tardieu et al. 2021; Chenu et al. 2017). Therefore, CGMs can be designed to provide a CGM-G2P framework, based on symbolic prior knowledge from plant and crop physiology and crop agronomy investigations. When aligned with the RPG and TPE context, the CGM-G2P framework can be used to investigate the genetic architecture of traits and for WGP applications (Fig. 1; Cooper et al. 2002, 2020, 2021b; Chapman et al. 2003; Campos et al. 2004; Hammer et al. 2006; Messina et al. 2006, 2018; Chenu et al. 2009; Technow et al. 2015; Diepenbrock et al. 2022).

**Fig. 3:**
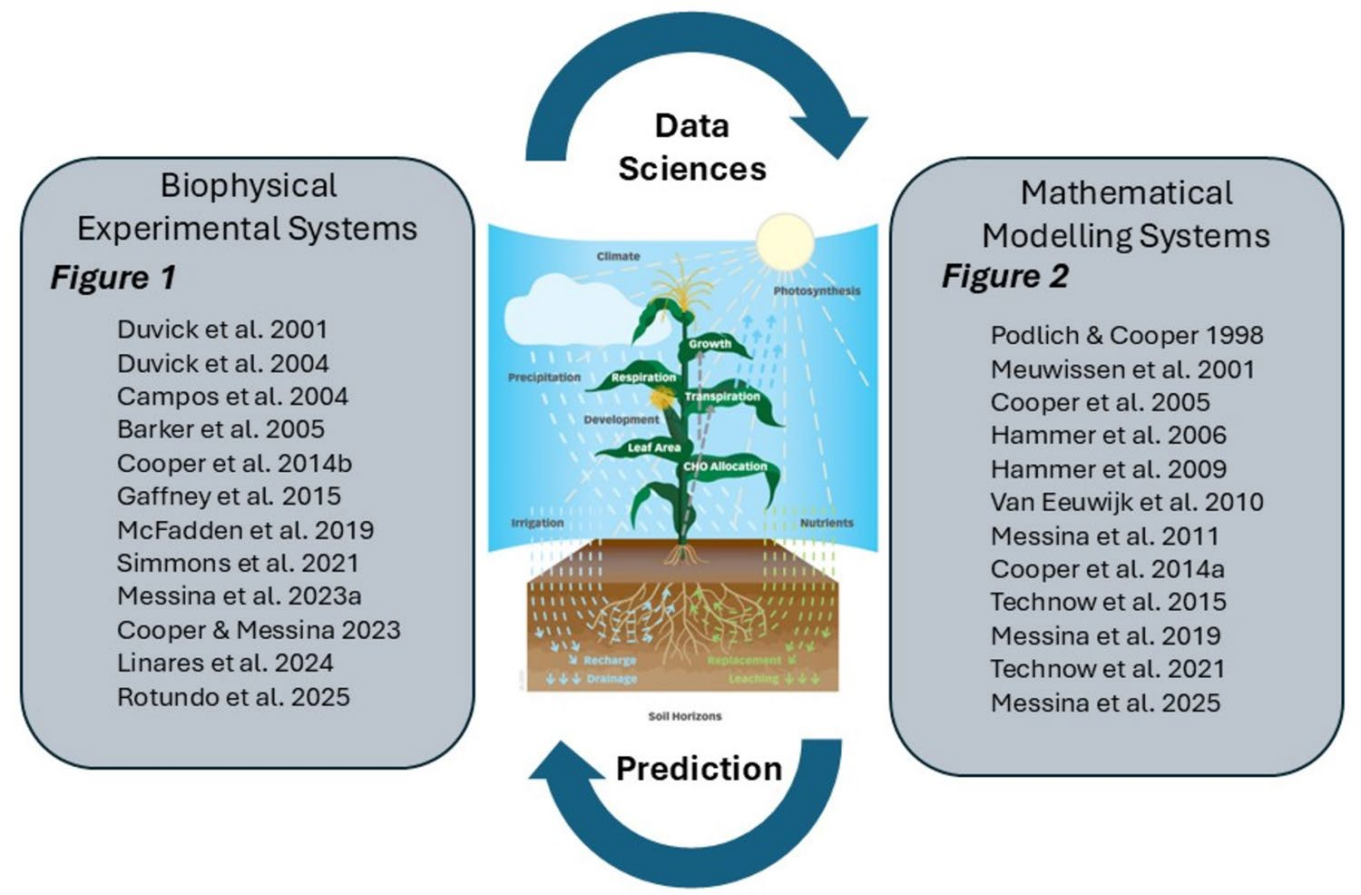
Schematic representation of the iterative research cycle involving integrated experimental and modelling systems applied to generate data to continually refine and update trait G2P models used for breeding prediction applications. We highlight the synergistic relationship between the overarching iterative experimental-modelling cycle. The cycle is focused on the operation of the breeding program within the biophysical target system (Fig. 1). The mathematical and modelling processes are applied to the data generated by the breeding program to enable predictions (Fig. 2). Two historical sequences of key publications, spanning more than two decades of continuous research, are included: (1) Biophysical experimental system; operating a maize breeding program within a target biophysical system to develop hybrids with improved yield and water productivity and continuously generate new data to test and refine the prediction models, and (2) Mathematical modelling system; developing and testing model-based predictions for breeding applications. The two sequences of key papers document a time series of an integrated experimental-modelling example, based on commercial maize breeding targeted to improve grain yield in drought-affected environments through manipulating crop water utilisation patterns and reproductive resilience. The publication sequences document the continuous operation of the iterative experimental-modelling cycle over multiple cycles of a breeding program

There are many statistical and AI-ML algorithms that can be applied to associate sequence variation with trait phenotypic variation to enable WGP using CGM-G2P models (Cooper et al. 2005; Yin et al. 2005; Hammer et al. 2006; Messina et al. 2011, 2018; Dong et al. 2012; Technow et al. 2015; Onogi et al. 2016; Millet et al. 2019; Toda et al. 2020; Jighly et al. 2023a,b; Wang et al. 2023; Kawakita et al. 2024). The algorithms are applied iteratively (Fig. 3; Cooper et al. 2002, 2020, 2021b; Hammer et al. 2006, 2019; Messina et al. 2009, 2025; Powell et al. 2025). The iterations involve cycles of model building and testing, operating between two general phases: (1) the CGM-G2P model development using sequence and trait phenotype data from relevant experiments, and (2) the model training and evaluation of the CGM-G2P predictions for any traits included in the CGM (Hammer et al. 2006, 2019; Messina et al. 2006, 2009, 2011, 2015, 2018, 2019, 2021, 2022, 2023a, 2025; Cooper et al. 2014b; Gaffney et al. 2015; Technow et al. 2015; Diepenbrock et al. 2022).

With appropriate attention to experimental design and representation of the RPG and TPE, all these iterative steps can be integrated into breeding program cycles to develop the CGM-G2P prediction framework (Fig. 1; Campos et al. 2004; Messina et al. 2009, 2011, 2025; Hammer et al. 2009; Cooper et al. 2014b; Cooper and Messina 2023; Escamilla et al. 2025). Continuous improvements of the CGM-G2P prediction framework over multiple breeding cycles was applied to accelerate breeding of maize hybrids with improved yield in drought-affected environments combined with improved yield potential (Campos et al. 2004; Barker et al. 2005; Messina et al. 2009, 2011, 2023a; Cooper et al. 2014b, 2020, 2023a; Gaffney et al. 2015; Diepenbrock et al. 2022; Cooper and Messina 2023).

Three key lessons for prediction-based breeding:

1. The CGM-G2P framework can be developed to broaden the context of trait physiology and QTL mapping studies to integrate prior understanding of traits and trait genetic architecture to accelerate breeding outcomes (Campos et al. 2004; Hammer et al. 2006; Cooper et al. 2021b; Tardieu et al. 2021; Cooper and Messina 2023; Escamilla et al. 2025).
2. Breeding context is still important for the CGM-G2P framework. If the objective is to accelerate genetic gain through improved breeding methodology, CGMs need to be developed to focus on the traits and their genetic architecture for the context of the RPG and TPE of the breeding program (Fig. 1; Campos et al. 2004; Cooper et al. 2014a,b; Messina et al. 2018, 2022, 2023a; Diepenbrock et al. 2022; Cooper and Messina 2023).
3. The development of a CGM-G2P framework for breeding applications must be done at scale within the context of the operations of the target breeding program (Campos et al. 2004; Messina et al. 2009, 2011, 2018, 2023a; Diepenbrock et al. 2022; Cooper and Messina 2023; Escamilla et al. 2025).

### Theme 4: How can ensembles of G2P models enhance WGP opportunities?

Themes 1, 2 and 3 provide background to studies of trait genetic architecture and applications to enhance prediction-based breeding (Table 1; Meuwissen et al. 2001; Campos et al. 2004; Bernardo and Yu 2007; Boer et al. 2007; Cooper et al. 2014a,b; Crossa et al. 2014, 2025; Messina et al. 2018; Cobb et al. 2018, 2019; Voss-Fels et al. 2019a; Gage et al. 2020; Diepenbrock et al. 2022; Varshney et al. 2021a,b; Jighly et al. 2023a,b; Escamilla et al. 2025). Based on experience from studying the genetic architecture of yield and related traits in maize, and the long-term efforts that underpin breeding maize hybrids with improved levels of drought tolerance (Duvick et al. 2004; Campos et al. 2004; Barker et al. 2005; Boer et al. 2007; Cooper et al. 2014a,b; Gaffney et al. 2015; Simmons et al. 2021; Messina et al. 2023a,b; Cooper and Messina 2023; Linares et al. 2023, 2024, 2025), we now consider some potential applications of AI-ML methods to facilitate prediction-based breeding. Our consideration of AI-ML methodology for prediction applications builds on the foundation of successful application of WGP for maize breeding applications (Campos et al. 2004; Cooper et al. 2014a,b; Gaffney et al. 2015; Diepenbrock et al. 2022; Messina et al. 2023b; Cooper and Messina 2023).

Applying the theoretical framework outlined by Messina et al. (2025), ensembles of heterogeneous prediction algorithms have the potential to create novel opportunities to tackle G2P dimensionality. We investigate the proposition of an advantage for WGP outcomes (Cooper et al. 2005; Ramstein et al. 2019; Powell et al. 2025; Messina et al. 2025). For traits of high G2P dimensionality, we can develop lines of investigation to explore how combinations of measurement and modelling methods can be applied as ensembles to investigate trait genetic architecture and enhance WGP (Fig. 1; Bian and Holland 2015; McCormick et al. 2021; Kick and Washburn 2023; Washburn et al. 2025; Tomura et al. 2025a; Kawakita et al. 2024; Messina et al. 2025). We illustrate this line of investigation through a case study of the trait of flowering time, based on the published results of the TeoNAM experiment (Fig. 2; Chen et al. 2019; Tomura et al. 2025a).

The TeoNAM data set was chosen to illustrate concepts related to using ensembles of prediction models because of its multi-parental design and the use of diverse parents (Chen et al. 2019). In summary, the TeoNAM design was implemented by developing recombinant inbred lines from five connected biparental crosses. The common parent for each cross in the TeoNAM was the public inbred maize line W22. The second parent for each cross was a teosinte line, representing the wild progenitor of domesticated maize. Thus, with the high level of genetic diversity exposed in each of the five crosses we can expect that many components of the trait genetic architecture (Table 1) for any trait will be segregating in the TeoNAM experiment. This high level of genetic diversity facilitates comparisons of the results of the investigations based on the statistical and AI-ML WGP models reported by Tomura et al. (2025a), with independent investigations providing prior knowledge of trait genetic architecture (Buckler et al. 2009; Dong et al. 2012; Chen et al. 2019; Wisser et al. 2019; Mayer et al. 2020). Prior investigations of the genetic architecture of flowering time traits of maize demonstrate that there is a network of gene interactions operating to determine the standing genetic variation for flowering time in any RPG (Dong et al. 2012). The dimensions of the gene network that determines the trait phenotype for individuals, that vary in any study, will depend on the diversity of the parents used to construct the mapping study. The TeoNAM mapping study represents extreme diversity allowing many of the G2P dimensions of flowering time expected in maize to be exposed for investigation. The extreme genetic diversity within the TeoNAM experiment provides a suitable basis for illustrating an application of the Diversity Prediction Theorem, Eq. (1), as a framework for evaluating the potential of ensembles for WGP applications to breeding (Fig. 1; Messina et al. 2025; Tomura et al. 2025a).

Tomura et al. (2025a) considered six WGP methods for their ensemble analysis of the TeoNAM data set (Figs. 1 and 2): ridge regression Best Linear Unbiased Prediction (rrBLUP), BayesB, Reproducing Kernel Hilbert Space (RKHS), Random Forest (RF), Support Vector Regression (SVR), Graph Attention Networks (GAT). They reported an advantage of the ensemble-based prediction, for both prediction accuracy and mean squared error, over the six individual WGP methods applied individually. Following the framework of Messina et al. (2025), they investigated the advantage of the ensemble prediction method over the individual prediction methods at two levels (Fig. 2a): (1) the diversity among the individual prediction methods for the predicted genotypic values, and (2) the diversity among the individual prediction methods for the weights of the SNP effects used to compute the genotypic values. They reported diversity in both the predicted genotypic values and the weights of the SNPs among the six prediction methods they considered. There was considerable heterogeneity among the 15 pairwise comparisons of the individual WGP methods (Fig. 2a). Some prediction methods were similar, and others showed greater divergence at both the genotypic and SNP prediction levels. Of particular interest was the strong divergence between the traditional linear statistical WGP models (rrBLUP and BayesB) and the AI-ML prediction models (RF, SVR and GAT). The semi-parametric RKHS prediction method differed from both the linear statistical and the AI-ML prediction models. Therefore, their ensemble prediction was a result of a weighted combination of the diverse G2P associations achieved by each individual prediction method. Importantly the ensemble generated a different G2P association for prediction than any that was achieved by an individual prediction method (Fig. 2b; Tomura et al. 2025a). Thus, novel features of the G2P association were revealed by the ensemble of prediction methods.

The results of Tomura et al. (2025a) provide an entry for further investigations of ensemble prediction methods. Their consideration of the diversity of G2P associations among prediction methods, as a basis for the ensemble advantage, aligns with expectations of the theoretical framework of Messina et al. (2025).

#### Theme 4.1: novel views of G2P dimensionality are revealed by ensemble analysis

We extend the results reported by Tomura et al. (2025a) by constructing a new graphical view applying circos plots (Fig. 2b; Krzywinski et al. 2009; Tomura et al. 2025b) to facilitate comparisons between their WGP results with the QTL mapping results reported by Chen et al. (2019) and the prior knowledge of the gene regulatory network for maize flowering reported by Dong et al. (2012). The circos plot graphical view contains a sequence of circular plots, with each ring displaying results for the 10 chromosomes of the maize genome. Starting from the outer ring and working inwards. The outermost ring provides a graphical view of the relative weights of the SNP contributions to the predicted genotypic values for the ensemble. The next six rings provide the relative weights of the SNP contributions to the predicted genotypic values individually for the six WGP methods contributing to the ensemble: rrBLUP, BayesB, RKHS, RF, SVR, and GAT. The next two rings provide the maize genome positions for the genes and QTL reported by Dong et al. (2012) as contributing to the seven components they distinguished for the gene regulatory network controlling flowering time of maize genotypes; Light Transduction, Circadian Clock, Photoperiod transduction pathway, Autonomous pathway, Aging pathway, Gibberellic Acid (GA) pathway, and the Pathway Integrator. The innermost ring gives the positions of the QTL for the flowering trait days to anthesis (DTA) that were identified by Chen et al. (2019) from their analysis of the TeoNAM data set. Within the centre of the graphical view, lines are used to connect SNP genome positions to indicate evidence for interactions identified between SNPs. To identify these putative QTL-by-QTL interactions, the strongest 0.005% Shapley Scores from the Random Forest analysis conducted by Tomura et al. (2025a) were used to identify putative SNP-by-SNP interactions (Tomura et al. 2025b). The red lines are used to identify putative cis-acting SNP interactions that occur between regions within a chromosome. The blue lines are used to identify putative trans-acting SNP interactions between chromosomes.

The putative SNP-by-SNP interactions (Fig. 2b; Tomura et al. 2025b) can be used to construct adjacency matrices and apply the methods of graph theory to enable gene network analysis of trait genetic architecture (Dorogovtsev and Mendes 2003; Cooper et al. 2005) and model their potential influences on any predictions used to implement selection (Podlich et al. 2004; Cooper et al. 2005; Technow et al. 2021; Powell et al. 2022).

The relative weights of the SNPs for the six WGP methods varied among the individual prediction models (Fig. 2b). However, some common genome regions consistently featured as the stronger contributors to multiple WGP models, e.g. the darker regions identified on Chromosomes 1, 6, 8, 9 and 10. Some of these regions also aligned with QTL identified by Chen et al. (2019) and also with the genes and QTL contributing to the flowering network of Dong et al. (2012), e.g. on Chromosomes 8 and 10. Further, the analysis of the Shapley scores from the Random Forest G2P analysis strongly indicated evidence of interactions between common genomic regions emphasised by multiple WGP models and the QTL identified by Chen et al. (2019). For example, there was strong evidence for interactions between SNPs within regions on chromosomes 8 and 10. For both regions, putative cis-acting (red lines) and trans-acting regions (blue lines) were identified. The region identified on chromosome 10 contains the known gene *ZmCCT10* (Dong et al. 2012; Stephenson et al. 2019) involved in photoperiod response and the region on chromosome 8 contains the known integrator gene *ZCN8* (Dong et al. 2012; Meng et al. 2011). Both regions were also identified as QTL in other studies of flowering time in maize (Buckler et al. 2009; Dong et al. 2012; Wisser et al. 2019; Mayer et al. 2020). While a detailed treatment of all the putative QTL-by-QTL interactions is not the purpose here, this highlighted example provides evidence that there is significant prior knowledge of the flowering time gene regulatory network that indicates the importance of biological interactions between *ZmCCT10* and *ZCN8* in the TeoNAM study (Chen et al. 2019) that can have important implications for trait WGP.

The results of the TeoNAM analyses by Tomura et al. (2025a) demonstrated a prediction advantage of ensemble-based methods. The circos plot graphical view presented here (Fig. 2; Tomura et al. 2025b), and the theoretical framework of Messina et al. (2025) provide a foundation for investigating the G2P dimensionality of trait genetic architecture in large scale breeding experiments (Fig. 1; Podlich et al. 2004; Cooper et al. 2005, 2014a,b; Messina et al. 2009, 2011; Technow et al. 2021). They also motivate consideration of an ensemble-based framework to develop and optimise WGP models for breeding applications (Messina et al. 2025).

We summarise key lessons for the breeding context that are drawn from our review of background technologies, experience working through the life cycles of prediction methodology development and outcomes from evaluation of predictive breeding technologies for commercial maize breeding:

1. Selection history, the RPG and TPE all combine to determine the “breeding context” within which WGP models can capture G2P dimensions of trait genetic architecture (Fig. 1; Table 1; Duvick et al. 2004; Campos et al. 2004; Cooper et al. 2014a; Diepenbrock et al. 2022; Escamilla et al. 2025).
2. The genetic architecture of the traits and the associated standing genetic variation targeted in breeding programs has high G2P dimensionality that can be targeted and harnessed to accelerate genetic gain (Fig. 1; Table 1; Messina et al. 2018, 2023a; Diepenbrock et al. 2022; Cooper and Messina 2023).
3. Experiments designed to sample contrasting envirotypes from the TPE and expose diverse G2P dimensions of trait genetic architecture for the RPG of the breeding program can create opportunities to exploit a larger range of the potential breeding trajectories (Fig. 1; Campos et al. 2004; Messina et al. 2011, 2018, 2023a; Cooper et al. 2014a,b; Technow et al. 2021; Diepenbrock et al. 2022; Choquette et al. 2023; Polzer et al. 2025; Amin et al. 2025).
4. Individual G2P models for WGP can enable acceleration of genetic gain for yield and related traits (Cooper et al. 2014a,b; Voss-Fels et al. 2019a; Messina et al. 2023a; Escamilla et al. 2025). However, each implementation can be expected to expose a subset of the potential breeding trajectories accessible from the standing genetic variation (Podlich et al. 2004; Cooper et al. 2005; Technow et al. 2021; Polzer et al. 2025). Diverse sets of G2P models for WGP can be used to create ensembles of models to investigate the contributions of different G2P dimensions of trait genetic architecture within the context of the standing genetic variation of a breeding program (Fig. 2; Tomura et al. 2025a; Messina et al. 2025).

## Emerging elements of novel prediction targets for applications of AI-ML in breeding

Following the theoretical framework of Messina et al. (2025) and the ensemble results from application of the framework to the TeoNAM example (Fig. 2; Chen et al. 2019; Tomura et al. 2025a), we hypothesise that for cases where the standing genetic variation for quantitative traits is determined by a genetic architecture that includes gene and trait networks that operate in a hierarchical G2P map, ensembles of prediction models that include combinations of symbolic biological and sub-symbolic statistical and AI-ML models can be designed to account for the high dimensionality of the trait G2P map to enhance WGP for targeted breeding applications (Cooper et al. 2005; Hammer et al. 2006; Messina et al. 2011, 2023a; Technow et al. 2015). We also interpret that in part the success of the early applications of the CGM-WGP methodology to accelerate maize breeding for improved yield and drought performance was an outcome of a long-term breeding effort applying prediction algorithms that leveraged prior knowledge of key trait networks that were captured by the maize CGM and the opportunities indicated by the G2P diversity framework of Messina et al. (2025) (Campos et al. 2004; Messina et al. 2009, 2011, 2023b; Cooper et al. 2014b; Gaffney et al. 2015; Cooper and Messina 2023). We can highlight some important elements for consideration in the design of novel prediction methods that leverage ensembles of diverse G2P models:

1. Diverse information on the associations between measures of genome diversity and trait phenotypic diversity obtained from applying different G2P prediction algorithms has the potential to emphasise different aspects of trait G2P dimensionality for investigation of trait genetic architecture and applications to prediction-based breeding methods.
2. As trait non-additivity increases in a particular breeding context, as emergent consequences of gene and trait networks, mapping and WGP studies designed to expose different dimensions of trait G2P dimensionality may identify alternative and novel pathways for crop genetic improvement (Campos et al. 2004; Cooper et al. 2005; Messina et al. 2011, 2022; Technow et al. 2021; Diepenbrock et al. 2022; Cooper and Messina 2023).
3. Experiments that are designed to expose novel trait G2P dimensionality may be appropriate targets for application of AI-ML interaction first models (e.g., Fig. 2, RKHS, RF, MLP, SVR, GAT; Powell et al. 2022, 2025; Wang et al. 2023; Negus et al. 2024; Crossa et al. 2025; Tomura et al. 2025a) versus the more traditional main-effects first models of quantitative genetics that treat the interactions as residuals (e.g., Fig. 2, GBLUP, Bayes Alphabet; Meuwissen et al. 2001; Bernardo and Yu 2007; Tomura et al. 2025a). Huang and Mackay (2016) clarified how these contrasting approaches for modelling allele effects and effects of allele substitution for QTL can reveal different modes of gene action and thus their importance for prediction applications. Cheverud and Routman (1995), using a two gene system, demonstrated how conditional additive genetic variance for the main-effects first models emerges in the presence of epistasis. Podlich et al. (2004) and Technow et al. (2021) demonstrated how such conditional additive genetic variance can emerge for more complex genetic networks under the influences of selection.
4. Ensembles of G2P prediction models applying combinations of both main-effects first and interaction first models provide a practical approach for investigating and using these different models of QTL effects for both discovery and prediction (Huang and Mackay 2016; Tomura et al. 2025a; Powell et al. 2025; Messina et al. 2025). The different weighting of the G2P models for prediction applications, as demonstrated in the ensemble results of Tomura et al. (2025a), has the potential to create new opportunities to identify novel pathways for crop breeding (Podlich et al. 2004; Cooper et al. 2005; Technow et al. 2021; Messina et al. 2023a; Linares et al. 2023, 2024, 2025).
5. Experimental design, high-throughput genomics, phenomics and enviromics, and analysis methods that expose different aspects of trait G2P dimensionality, each open new opportunities for the breeder to decide how to weight and borrow different sources of G2P information for discovery and prediction algorithms (Figs. 1 and 2; Campos et al. 2004; van Eeuwijk et al. 2019; Diepenbrock et al. 2022; Washburn et al. 2025; Tomura et al. 2025a; Powell et al. 2025; Sangjan et al. 2025).
6. An ability to focus discovery efforts on the G2P properties revealed through methods such as ensembles provides a foundation to target QTL and gene discovery at the genome sequence level. The interconnections between the genome regions discovered can also be exposed for further investigation of gene and trait networks to enhance prediction using AI-ML (Fig. 2).

## Perspectives and future directions

The dimensionality of the G2P hierarchy for the target traits of crop breeding programs is vast (Table 1; Campos et al. 2004; Cooper et al. 2005; Hammer et al. 2006; Reynolds and Langridge 2016; Ramstein et al. 2019; Kholová et al. 2021; Messina et al. 2011, 2022; Diepenbrock et al. 2022; Washburn et al.^2025^). Here we have provided some perspectives on how to iteratively tackle the high G2P dimensionality that are drawn from technology development and maize breeding experience (Fig. 3). Motivations are to continually seek advances in our experimental design and data generation processes, G2P modelling methodologies and prediction theory for crop breeding that consider the implications of gene networks for trait genetic architecture (Podlich and Cooper 1998; Cooper et al. 2002, 2005; Hammer et al. 2006; Messina et al. 2009, 2011, 2018, 2023a, 2025; Marjoram et al. 2014; Boyle et al. 2017; Technow et al. 2021; Voss-Fels et al. 2019a; Powell et al. 2025). Quantitative genetics’ theory often ignores these implications or assumes a stationary additive approximation for allele effects under the influences of selection in breeding programs. This can work for many short-term breeding considerations (Fig. 1; one or a few breeding cycles). Further, simulation and experimental investigations focused on long-term genetic gain considerations for commercial maize breeding demonstrate that WGP methods that do not explicitly incorporate such prior knowledge of gene and trait networks can still identify opportunities to accelerate genetic gain for many situations faced by breeding programs (Duvick et al. 2004; Cooper et al. 2005, 2014a,b; Technow et al. 2021; Messina et al. 2023a). However, these approaches continue to rely heavily on updating MET and associated data sets and G2P model retraining (Podlich et al. 2004; Cooper et al. 2005, 2020; Technow et al. 2021; Polzer et al. 2025; Escamilla et al. 2025). We argue that the limited capacity of our current empirical breeding strategies and WGP methods to explicitly incorporate the growing body of prior knowledge of the many consequences of such biological interactions is an opportunity for targeted AI-ML research to realise the full potential of genetic diversity for long-term genetic gain (Cooper et al. 2005; Podlich et al. 2004; Messina et al. 2011, 2025; Technow et al. 2021; Powell et al. 2021, 2022, 2025; Linares et al. 2023, 2024, 2025; Escamilla et al. 2025). Here AI-ML technologies can add new capabilities to the prediction toolkit of the breeder (Messina et al. 2025).

Without specific modelling of breeding context, it is difficult to navigate from individual gene and allele effects studied at the molecular level to crop yield responses for a TPE (Fig. 1; Campos et al. 2004; Cooper et al. 2005; Messina et al. 2011; Tardieu 2012; Marjoram et al. 2014; Ramstein et al. 2019; Linares et al. 2023, 2024, 2025; Khaipho-Burch et al. 2023; Escamilla et al. 2025). We illustrated some aspects of the high dimensionality of the G2P system using the developmental trait of time to flower for the TeoNAM study (Fig. 2; Chen et al. 2019; Tomura et al. 2025a). The features of trait genetic architecture illustrated using the TeoNAM example are expected to apply for many quantitative traits that are the targets of breeding programs (Buckler et al. 2009; Guo et al. 2014; Mayer et al. 2020; Wisser et al. 2019; Technow et al. 2021; Messina et al. 2023a,b; Cooper and Messina 2023; Powell et al. 2025; Linares et al. 2023, 2024, 2025). We note that the diversity of the TeoNAM experiment is extreme relative to the genetic diversity that is the target of many breeding programs (Cooper et al. 2014b; Messina et al. 2023a). However, the genetic diversity that is targeted by breeding programs is a continuum, depending on many factors including crop, history of breeding, the complexity of the TPE, and resources available to the breeding program (Duvick et al. 2001, 2004; Kholová et al. 2021; Messina et al. 2022; Choquette et al. 2023; Werner et al. 2025; Polzer et al. 2025; Escamilla et al. 2025).

Reflecting on progress from long-term breeding efforts, for the complex quantitative traits and mechanisms contributing to yield potential and yield under abiotic stresses (e.g., drought, high temperatures, high atmospheric vapour pressure deficit) our current gene discovery strategies are resource intensive and they have had a low success rate in translating many of the discoveries at the genome sequence level into contributions to improved yield and yield stability and accelerated genetic gain (Campos et al. 2004; Simmons et al. 2021; Cooper and Messina 2023; Khaipho-Burch et al. 2023; Linares et al. 2025). The perspective we bring to this translation challenge is that for important quantitative traits in crop breeding, such as grain yield, our gene discovery paradigm and toolkit of methods has to date been effective in revealing important details of trait genetic architecture and trait biology at lower levels in the hierarchies of G2P trait architecture, while it has had limited impact in delivering consistent predictability at the higher levels of the trait G2P hierarchy to deliver accelerated rates of genetic gain (Campos et al. 2004; Guo et al. 2014; Voss-Fels et al. 2019a; Simmons et al. 2021; Khaipho-Burch et al. 2023; Linares et al. 2023, 2024, 2025). In some cases, the discovered gene efficacy delivers an acceptable level of predictability close to the discovery context (Campos et al. 2004; Simmons et al. 2021; Linares et al. 2023, 2024, 2025). However, the predictability diminishes to unacceptable levels for the majority of the GxExM state space of the target crops and agricultural systems that are beyond the discovery context (Campos et al. 2004; Guo et al. 2014; Powell et al. 2021; Linares et al. 2023, 2024, 2025; Cooper et al. 2023a).

There will always be challenges for prediction-based breeding. However, we have extensive prior knowledge of trait genetic architecture and now have an emerging theory and toolkit of symbolic and sub-symbolic modelling methods to enable prediction that includes many important interactions that exist within the gene and trait networks (Campos et al. 2004; Hammer et al. 2006; von Rueden et al. 2023; Messina et al. 2025). The methods of AI-ML will be an important component of the toolkit. Early applications of these prediction-based methods to maize breeding have demonstrated improved rates of genetic gain for yield and yield stability that can account for important dimensions of the G2P hierarchy and GxExM interactions (Cooper et al. 2014a,b, 2020; Technow et al. 2015; Diepenbrock et al. 2022; Messina et al. 2018, 2022, 2023a; Cooper and Messina 2023).

Three key lessons for prediction-based breeding:

1. Breeding context and scale are important for investigations of trait genetic architecture (Fig. 1; Table 1; Campos et al. 2004; Cooper et al. 2014a; Escamilla et al. 2025). The theory and experimental results to date indicate that there is unlikely to be a “free lunch” (Wolpert and Macready 1997) from applications of AI-ML to “big data” for predictive breeding. Instead, we need to better understand the strengths and weaknesses of AI-ML technologies for predictive breeding applications. The “diversity prediction theorem” (Page 2010, 2018; Messina et al. 2025) provides a framework for such investigations and encourages the application of ensemble-based modelling approaches (Fig. 2; Tomura et al. 2025a).
2. Utilising the genomics, phenomics, and enviromics data that currently can be collected around the operations of breeding programs can be expected to provide a continuum of small to large opportunities to use AI-ML to improve the efficiency of breeding strategies and accelerate the current trajectories of crop improvement (Fig. 1; Voss-Fels et al. 2019a; Cooper and Messina 2023; Messina et al. 2023a; Sangjan et al. 2025; Escamilla et al. 2025).
3. To discover and leverage the full range of opportunities from potential applications of AI-ML to enhance predictive breeding, future considerations for “big projects” need to be focussed on the design of large-scale experiments and transdisciplinary teams to test alternative breeding strategies for novel crop improvement targets (Wisser et al. 2019; Diepenbrock et al. 2022; Messina et al. 2022; Choquette et al. 2023; Werner et al. 2025; Polzer et al. 2025). This will require complementary experimental and simulation comparisons of proposed prediction-based breeding methods for the breeding context of the current breeding strategies conducted over multiple cycles (Fig. 3; Messina et al. 2011, 2023a; Bernardo 2020; Technow et al. 2021; Powell et al. 2025; Polzer et al. 2025). These investigations require new research strategies that iteratively integrate experimental and simulation capabilities within the “breeding context” (Fig. 3; Podlich and Cooper 1998; Cooper et al. 2002, 2005, 2014a,b; Campos et al. 2004; Messina et al. 2009, 2011, 2025; Bernardo 2020; Technow et al. 2021).

## Data Availability

All TeoNAM datasets utilised for the example included in this review were collected by Chen et al. (2019). Phenotype data are publicly available at panzea/genotypes/GBS/TeosinteNAM and marker data are available at panzea/genotypes/GBS/TeosinteNAMrespectively. The code generated for this experiment is shared at https://github.com/ShunichiroT/ensemble.
